# Siltuximab for the treatment of early complications after chimeric antigen receptor T-cell therapy for acute lymphoblastic leukemia in children, adolescents, and young adults

**DOI:** 10.1186/s40164-025-00638-3

**Published:** 2025-04-02

**Authors:** Víctor Galán-Gómez, Berta González-Martínez, Anna Alonso-Saladrigues, Susana Rives, Blanca Herrero, Mi Kwon, Jose Sánchez-Pina, Jordi Minguillón, Isabel Martínez-Romera, Isabel Mirones Aguilar, Carmen Mestre-Durán, Gema Casado, María Sánchez-Martín, Carlos Echecopar, Carlos González-Pérez, Odelaisy León-Triana, Cristina Aguirre-Portolés, Águeda Molinos-Quintana, Pere Barba, Pascual Balsalobre, Antonio Pérez-Martínez

**Affiliations:** 1 Pediatric Hemato-Oncology Department, La Paz University Hospital, Paseo de La Castellana, 261, 28046 Madrid, Spain; 2 Leukemia and Lymphoma Department, Pediatric Cancer Center Barcelona (PCCB), Hospital Sant Joan de Déu, Barcelona, Spain; 3 CIBERER-ISCIII, Sant Joan de Déu Research Institute, Barcelona, Spain; 4 Pediatric Hemato-Oncology Department, Pediatric University Hospital del Niño Jesús, Madrid, Spain; 5 Hematology and Hemotherapy Department, Gregorio Marañón Health Research Institute, General University Hospital Gregorio Marañón, Madrid, Spain; 6 Hematology and Hemotherapy Department, University Hospital 12 de Octubre, Madrid, Spain; 7 CIBERER-ISCIII, IdiPAZ-CNIO Pediatric Onco-Hematology Clinical Research Unit, Madrid, Spain; 8 Advanced Therapy Medicinal Products Production Unit, La Paz University Hospital, Madrid, Spain; 9 Pharmacy Department, La Paz University Hospital, Madrid, Spain; 10 Pediatric Intensive Care Department, La Paz University Hospital, Madrid, Spain; 11 Biostatistics Unit, Institute for Health Research (IdiPAZ), Madrid, Spain; 12 Hematology Department, Hospital Virgen del Rocío, Seville, Spain; 13 Hematology Department, Vall d’Hebron University Hospital, Autonomous University of Barcelona, Barcelona, Spain; 14 Spanish Group for Hematopoietic Transplantation and Cellular Therapy (GETH-TC), Madrid, Spain; 15 Pediatric Department, Autonomous University of Madrid, Madrid, Spain; 16 Advanced Therapies Mixed Unit, CIEMAT/IIS-FJD, Madrid, Spain

**Keywords:** Tocilizumab, Siltuximab, Chimeric antigen receptor—CAR, Cytokine release syndrome, Immune effector cell-associated neurotoxicity syndrome—ICANS, Neurotoxicity, Leukemia

## Abstract

**Background:**

Cytokine release syndrome (CRS) and immune effector cell-associated neurotoxicity syndrome (ICANS) are complications associated with CAR T-cell therapy. Siltuximab directly binds interleukin-6 (IL-6) and may be safe and effective as first-line therapy for CRS or ICANS.

**Methods:**

A retrospective study was conducted on pediatric, adolescent and young adult (AYA) patients treated with siltuximab after CAR T-cell therapy for B-ALL.

**Results:**

A total of 118 patients treated were included: 97 patients developed CRS (82%), and 26 patients (22%) developed ICANS. Sixty-five of those that developed CRS (55%), received treatment. In 46/65 (71%), tocilizumab was administered as anti-IL-6 drug, and 19/65 (29%) patients received siltuximab to treat tocilizumab-refractory CRS (n = 13, 68%), or as first-line CRS treatment (n = 6, 32%). Nine patients treated with siltuximab (47%) developed ICANS. With a median follow-up of 12.1 months, 7 patients remained alive.

**Conclusions:**

To the best of our knowledge, we present the largest reported cohort of patients treated with siltuximab for CRS following CAR T-cell therapy for B-ALL. Siltuximab’s safety profile and its inhibition of IL-6 effects suggest that it should be investigated as first-line therapy in prospective clinical trials.

**Supplementary Information:**

The online version contains supplementary material available at 10.1186/s40164-025-00638-3.

## Background

Although chimeric antigen receptor (CAR) T-cell therapy has improved the outcome of relapsed/refractory (r/r) B-cell acute lymphoblastic leukemia (B-ALL), the specific drug management of emerging toxicities, such as cytokine release syndrome (CRS) and immune effector cell-associated neurotoxicity syndrome (ICANS), increases the risk of non-relapse mortality [1]. IL-6 is a key cytokine involved in the pathogenesis of CRS and likely in the pathogenesis of ICANS after CAR T-cell therapy [2–4]. Tocilizumab and siltuximab are both monoclonal antibodies targeting the IL-6 receptors and soluble IL-6, respectively, and are critical drugs in the management of CRS and ICANS after CAR T-cell therapy. However, there are notable differences between them in terms of their mechanism of action, pharmacokinetics, and clinical use, as siltuximab is not indicated for the first-line management of acute complications secondary to CAR T-cell therapy. Tocilizumab binds to both, soluble and membrane-bound IL-6 receptors, and has a shorter half-life (t1/2: 5–13 days), requiring more frequent dosing of 8 mg/kg (12 mg/kg for patients under 30 kg), with a median of 1–4 doses. Tocilizumab clinical indications include rheumatoid arthritis, juvenile idiopathic arthritis, and CRS induced by CAR T-cell therapy [5]. Siltuximab directly binds to soluble IL-6, inhibiting its binding to IL-6 receptors. The pharmacokinetic profile supports a single dose of 11 mg/kg and exhibits a longer half-life (t1/2: 12–20 days) with a standard dosing interval of every three weeks. It is primarily approved for the treatment of Castleman’s disease [6, 7]. Clinical data on the use of siltuximab for CRS after CAR T-cell therapy are scarce and mostly focused on adult patients [8]. Some early clinical trials and case reports have suggested that siltuximab can mitigate CRS severity and reduces the need for additional therapies in certain patients with refractory CRS and ICANS [8–12].

There is limited clinical experience with siltuximab in CRS and ICANS induced by CAR T-cell therapy in clinical trials and real-world studies, which could determine an alternative dosing regimen for this drug different from that established for Castleman’s disease. Thus, more evidence is needed to better define the role of siltuximab in CRS and/or ICANS management. This work retrospectively analyzes the experience of the use of siltuximab in the management of CRS after two CAR T-cell therapies: tisagenlecleucel and tandem CD19/CD22, the latter as a compassionate use therapy in a cohort of children and AYA diagnosed with r/r B-ALL.

## Methods

### Study design and data source

We hereby present the results of a national retrospective observational study aimed at assessing the experience, outcomes and safety of pediatric and AYA patients treated with siltuximab for complications induced by CAR T-cell therapy, specifically tisagenlecleucel (Kymriah®) under commercial approval, and tandem CD19/CD22 CAR T-cell therapy as a compassionate use program for those patients with refractory or relapsed disease not eligible for tisagenlecleucel. The data from patients treated with tisagenlecleucel were extracted from the anonymized, electronic and mandatory CAR T-cell Spanish registry from Spanish Group for Hematopoietic Stem Cell Transplantation and Cellular Therapy (GETH-TC). Data related to patients that received treatment with tandem CD19/CD22 CAR T-cell were obtained from an internal record of the CAR T-cell activity from La Paz University Hospital, a tertiary hospital from Madrid, Spain.

### Patients

Clinical and demographical data were collected from all patients aged 0 to 25 who were treated with tisagenlecleucel CAR T-cell therapy for r/r B-ALL at 5 Spanish centers between January 2019 and December 2023. Same data were collected for patients treated at La Paz University Hospital for patients with r/r or otherwise therapy-inaccessible B-ALL treated with tandem CD19/CD22 CAR T-cell therapy (Miltenyi Biomedicine) under a compassionate use between September 2020 and April 2024. Focus was placed on the data concerning patients who received siltuximab as part of the CRS management.

### Chimeric antigen receptor T-cell products, lymphodepleting regimen and CAR T-cell related toxicities

Patients received tisagenlecleucel as part of a clinical approval indication or academic tandem CD19/CD22 CAR T-cell therapy [13] under a compassionate use program if not eligible for tisagenlecleucel. The lymphodepleting regimen was based on fludarabine and cyclophosphamide. The tumor burden (TB) prior to the lymphodepleting regimen was reported as the percentage of CD19^+^ leukemia blasts [14, 15] by morphology and/or flow cytometry in a pre-lymphodepletion bone marrow sample. Patients were monitored according to the CRS and ICANS grading scale developed by the American Society for Transplantation and Cellular Therapy (ASTCT) [16].

For patients treated with tisagenlecleucel, CRS and ICANS were managed as recommended by the current consensus guidelines, employing siltuximab as a rescue drug for refractory CRS or in case of ICANS with concomitant CRS treatment [14]. For patients treated with tandem CD19/CD22 CAR T-cell, siltuximab was used as a first-line therapy in CRS management as a multiprofessional medical decision because of the patient’s high risk of CRS/ICANS development, by avoiding the IL-6 peak after tocilizumab administration [2, 17, 18]. In three specific cases, and always prioritizing patient well-being, the relevant multidisciplinary committee decided to modify the standard treatment (patients number 10, 14 and 15). Refractory CRS was defined as CRS that persisted or worsened despite the adequate administration of CRS first-line treatment.

Other different lines of treatment were documented, as well as the use of additional immunosuppressive drugs (steroids, anakinra) for CAR T-cell toxicity management. The day of onset, duration and severity grade of both CRS and ICANS were also recorded.

Delayed immune effector cell-associated hematotoxicity (ICAHT) was defined as a platelet count < 100 × 10^9^/L and/or absolute neutrophil count < 1 × 10^9^/L at day + 28 after CAR T-cell infusion. Similarly, immune effector cell-associated hemophagocytic lymphohistiocytosis-like syndrome (IEC-HS) [19] and any documented viral, bacterial, or fungal infection were reported. Lastly, we included response to CAR T-cell therapy at day 28, outcome and last follow-up.

### Statistical analysis, variables, and endpoints

The primary endpoints of the study were to describe the main characteristics and clinical outcomes of patients treated with siltuximab compared to those who received tocilizumab. Statistical analyses were conducted using R software (R Core Team, 2024). Qualitative variables were summarized as absolute frequencies and percentages, while quantitative variables were reported as means with standard deviations or medians with interquartile ranges, depending on the distribution of the data. Group comparisons for categorical variables were performed using Fisher's exact test, whereas continuous variables were analyzed using either ANOVA or the Kruskal–Wallis test, based on their distribution. A p-value of < 0.05 was considered statistically significant. The probability of overall survival (OS) was estimated using the Kaplan–Meier method with a 95% confidence interval (95% CI). OS was defined as the time from CAR T-cell infusion to death from any cause. Endpoints were determined at the time of the last contact, loss to follow-up, or death.

## Results

### Patients, cytokine release syndrome and immune effector cell-associated neurotoxicity syndrome after CAR T-cell therapy

Data were retrieved from 118 children and AYA diagnosed with r/r B-ALL treated with CAR T-cell therapy: tisagenlecleucel (n = 108, 92%) or tandem CD19/CD22 CAR T-cell therapy (n = 10, 8%), in five Spanish tertiary centers between January 2019 and April 2024. A total of 97 (82%) patients developed CRS (37 grade 1, 30 grade 2, 19 grade 3, and 11 grade 4). ICANS was observed in 26 (22%) patients (5 grade 1, 5 grade 2, 11 grade 3, and 5 grade 4). Summary of the patients who received anti-IL-6 drugs for CRS management (n = 65, 67%) is shown in Table 1. They are divided into patients who only received tocilizumab (n = 46, 71%) and patients who received siltuximab for tocilizumab refractory CRS (n = 13, 20%) or as CRS first-line treatment (n = 6, 9%).

**Table 1:** Patient characteristics based on anti-IL-6 treatment

	CRS Management				
	Tocilizumab (N = 46)	Siltuximab tocilizumab refractory (N = 13)	Siltuximab first-line therapy (N = 6)	Total (N = 65)	p value
Age at CAR T-cell infusion (years)					0.483^1^
Median (IQR)	9.5 (13.4)	12.6 (6.8)	10.1 (12.6)	10.1 (12.3)	
Gender, n (%)					0.666^2^
MALE	21 (46%)	6 (46%)	4 (67%)	31 (48%)	
FEMALE	25 (54%)	7 (54%)	2 (33%)	34 (52%)	
Weight (kg)					0.366^1^
Median (IQR)	29.4 (32.4)	46.5 (19.2)	32.0 (10.6)	31.6 (27.6)	
B-ALL type, n (%)					0.015^2^
B-ALL NOS	26 (57%)	8 (62%)	0	34 (52%)	
B-common	10 (22%)	2 (15%)	6 (100%)	18 (28%)	
pre-B	8 (17%)	2 (15%)	0	10 (15%)	
pro-B	1 (2%)	1 (8%)	0	2 (3%)	
B-other	1 (2%)	0	0	1 (2%)	
Previous HSCT, n (%)					0.595^2^
No	28 (61%)	10 (77%)	4 (67%)	42 (65%)	
Yes	18 (39%)	3 (23%)	2 (33%)	23 (35%)	
Bridge treatment, n (%)					0.512^2^
Low intensity	24 (52%)	6 (46%)	3 (50%)	33 (50%)	
High intensity	12 (26%)	6 (46%)	1 (17%)	19 (29%)	
Inotuzumab	8 (17%)	1 (8%)	2 (33%)	11 (17%)	
Blinatumomab	1 (2%)	0	0	1 (2%)	
No bridge	1 (2%)	0	0	1 (2%)	
TB pre-lymphodepletion					0.178^1^
Median (IQR)	23.0 (52.3)	50.3 (77.3)	31.3 (69.1)	23.1 (59.8)	
CAR T-cell product, n (%)					< 0.001^2^
Tandem CD19/CD22	1 (2%)	1 (8%)	4 (67%)	6 (9%)	
Tisagenlecleucel	45 (98%)	12 (92%)	2 (33%)	59 (91%)	
CRS grade, n (%)					0.003^2^
1–2 (ASTCT)	30 (65%)	2 (15%)	4 (67%)	36 (55%)	
3–4 (ASTCT)	16 (35%)	11 (85%)	2 (33%)	29 (45%)	
Day of CRS onset					0.362^1^
Median (IQR)	1 (3)	1 (1)	1 (0)	1 (2)	
CRS resolution (days)					0.085^1^
Median (IQR)	6 (5)	9 (4)	5.5 (3.3)	6 (5)	
ICANS, n (%)					0.274^2^
No	33 (72%)	6 (46%)	4 (67%)	43 (66%)	
Yes	13 (28%)	7 (54%)	2 (33%)	22 (34%)	
ICANS grade, n (%)					0.158^2^
1–2 (ASTCT)	7 (54%)	1 (14%)	0	8 (36%)	
3–4 (ASTCT)	6 (46%)	6 (86%)	2 (100%)	14 (64%)	
Days of ICANS onset					–
Median (range)	8 (5–12)	6 (5–8)	10.5 (8–13)	7.5 (5–13)	
ICANS duration (days)					–
Median (range)	5 (1–16)	4 (2–21)	5.5 (4–7)	4.5 (1–21)	
Delayed ICAHT, n (%)					0.077^2^
No	23 (50%)	2 (15%)	3 (50%)	28 (43%)	
Yes	23 (50%)	11 (85%)	3 (50%)	37 (57%)	
Infections, n (%)					0.825^2^
No	34 (74%)	9 (69%)	4 (67%)	47 (72%)	
Yes	12 (26%)	4 (31%)	2 (33%)	18 (28%)	
IEC-HS					0.105^2^
No	40 (87%)	11 (85%)	3 (50%)	54 (83%)	
Yes	6 (13%)	2 (15%)	3 (50%)	11 (17%)	
PICU admission, n (%)					0.042^2^
No	24 (52%)	2 (15%)	2 (33%)	28 (43%)	
Yes	22 (48%)	11 (85%)	4 (67%)	37 (57%)	
PICU length (days)					0.629^1^
Median (IQR)	7 (5)	6 (2.5)	4.5 (7)	6 (5.2)	
Response (D28), n (%)					0.049^2^
CR	20 (56%)	1 (11%)	3 (60%)	24 (48%)	
CRi (delayed ICAHT)	16 (44%)	8 (89%)	2 (40%)	26 (52%)	
Last status, n (%)					0.026^2^
Dead	17 (37%)	10 (77%)	2 (33%)	29 (45%)	
Alive	29 (63%)	3 (23%)	4 (67%)	36 (55%)	
Cause of dead, n (%)					0.155^2^
No-CRM	16 (94%)	8 (80%)	1 (50%)	25 (86%)	
CRM	1 (6%)	2 (20%)	1 (50%)	4 (14%)	
Alive and free of disease, n (%)					0.039^2^
Yes	27 (93%)	1 (33%)	4 (100%)	32 (89%)	
No	2 (7%)	2 (67%)	0	4 (11%)	

ASTCT, American Society for Transplantation and Cellular Therapy; B-ALL, B-cell acute lymphoblastic leukemia; CAR, chimeric antigen receptor; CR, complete remission; Cri, complete remission with incomplete count recovery, defined as platelet count < 100 × 10^9^/L and/or absolute neutrophil count < 1 × 10^9^/L. CRS, cytokine release syndrome; HSCT, hematopoietic stem cell transplantation; IEC-HS, Immune Effector Cell-Associated hemophagocytic lymphohistiocytosis like syndrome; ICAHT, Immune effector cell-associated hematotoxicity; ICANS, immune effector cell-associated neurotoxicity syndrome; PICU, pediatric intensive care unit; rCRS, tocilizumab refractory cytokine release syndrome; rICANS, steroid refractory immune effector cell-associated neurotoxicity syndrome; TB, tumor burden

^1^Kruskal–Wallis rank sum test

^2^Fisher’s Exact Test for Count Data

**Fig. 1:**
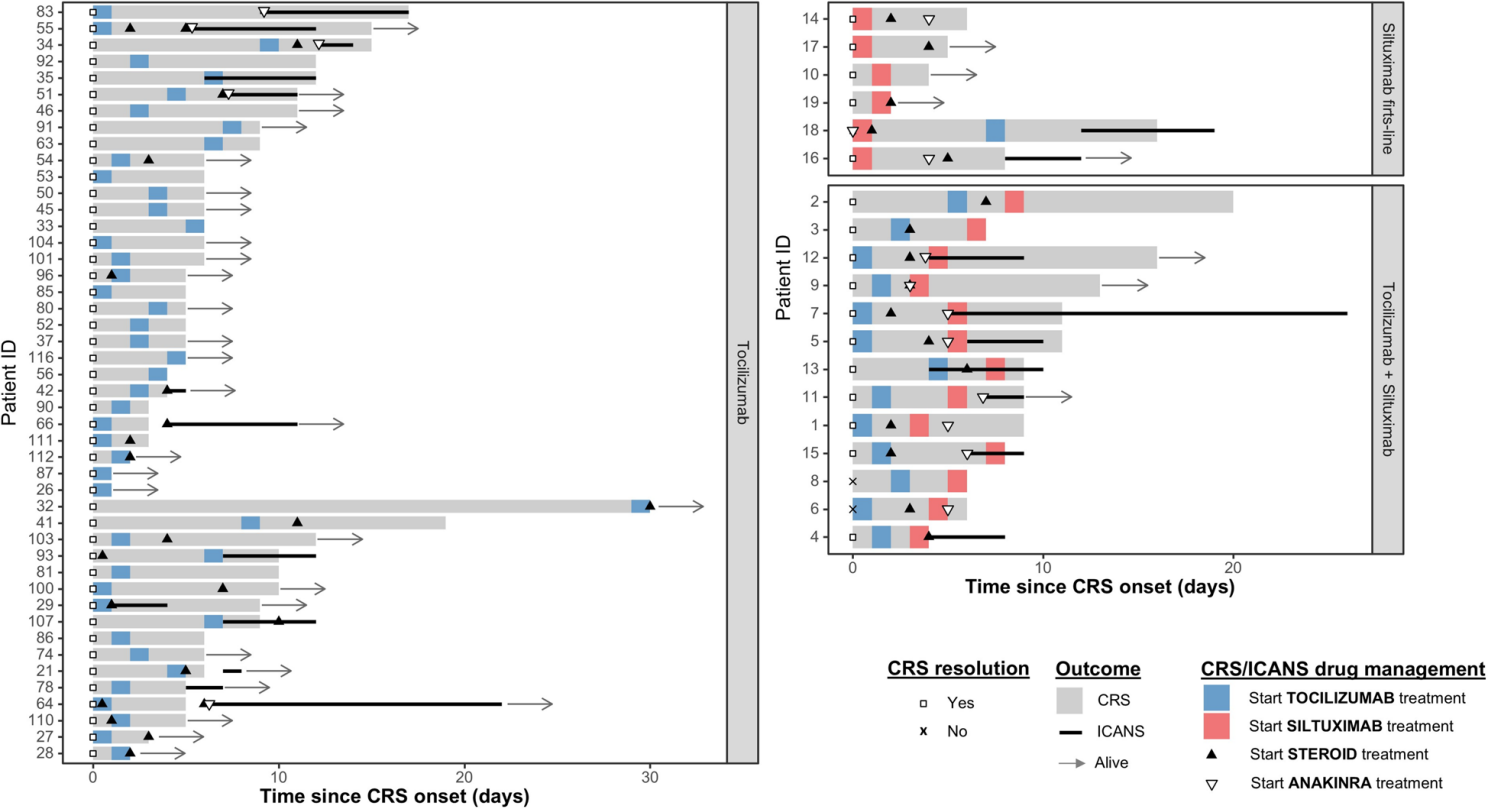
Swimmer plot of CRS/ICANS management and outcomes after CAR T-cell therapy

We now briefly describe the cohort of 19 patients who received siltuximab. Siltuximab was administered as a single dose (11 mg/kg) with a median of 5 days from CAR T-cell infusion (IQR 4) in 15/19 (79%) patients. An identical additional dose was administered on 4/19 (21%) before the 21-day period specified in the drug’s guidelines had elapsed because of CRS severity. TB median pre-lymphodepletion was higher in patients treated with siltuximab (Table 1). CRS severity (grade 3–4) was higher in those patients that were tocilizumab refractory (p < 0.05). Also, for this group of patients, the median weight was greater. The median time to CRS presentation after CAR T-cell infusion in all patients that received CRS treatment was 1 day (IQR 2). Thirteen (68%) patients needed more than 2 lines of treatment (Table 2, Fig. 1). CRS was resolved in all patients except for two in the tocilizumab refractory group. The median time to CRS resolution was 5.5 days (IQR 3.3) in the cohort of patients who received siltuximab as first-line treatment, and 9 days (IQR 4) in the cohort of patients who received siltuximab for tocilizumab refractory CRS. In the group of patients who received tocilizumab as first-line CRS treatment with a favorable CRS outcome, the median time to resolution was 6 days (IQR 5), not statistically significance.

**Table 2:** Patient treated with siltuximab characteristics, CRS/ICANS management with day of drug administration from CAR T-cell infusion (D) and outcome

Patient	B-ALL	CAR-T	TB (%)	CRS (day start–end, maximum grade)	CRS Treatment	ICANS (day start–end, maximum grade)	ICANS treatment	CRS/ICANS resolution	EVALUATION D + 28	OUTCOME (months)
1	Pre-B	T	5.5	2–11, G3	Tocilizumab (× 2, D2), MP, DXM (D4), siltuximab (× 1, D5), anakinra (D7)	No	No	Yes/-	CR, MRD-	DOD (4)
2	Pre-B	T	66	0–20, G2	Tocilizumab (× 2, D5), MP (D7), siltuximab (× 1, D8)	No	No	Yes/-	CR, MRD-	Loss of FU (3)
3	NOS	T	88	1–8, G2	Tocilizumab (× 3, D3), MP (D4), siltuximab (× 1, D7)	No	No	Yes/-	CR, MRD-	DOD (4)
4	NOS	T	23.3	1–5, G3	Tocilizumab (× 2, D2), siltuximab (× 1, D5)	5–9, G4	DXM (D5),	Yes/Yes	CR, MRD-	DOD (10)
5	NOS	T	10.7	0–11, G4	Tocilizumab (× 2, D0), MP (D4), siltuximab (× 2, D5, D6), anakinra (D5)	6–10, G3	MP (D6)	Yes/Yes	CR, MRD-	DOD (8)
6	NOS	T	50.3	1–7, G4	Tocilizumab (× 2, D1), MP (D4), siltuximab (× 2, D5, D6), MP-HD (D6), anakinra (D6)	No	No	No/-	-/MOF	CRM (7 days)
7	NOS	T	0.8	2–13, G4	Tocilizumab (× 2, D2), MP (D4), siltuximab (× 1, D7), anakinra (D7)	7–28, G2	MP (D7)	Yes / Yes	CR, MRD-	TRM (12)
8	Common	T	93	0–6, G4	Tocilizumab (× 2, D2), siltuximab (× 1, D5)	No	No	No/-	-/CRS	CRM (6 days)
9	Pro-B	T	15	1–14, G4	Tocilizumab (× 1, D2), MP (D4), siltuximab (× 1, D4), anakinra (D4)	No	No	Yes/-	PD	Alive, relapse (29)
10	Common	T	59	9–13, G2	Siltuximab (× 1, D10)	No	No	Yes/-	CR, MRD-	Alive, CR (27)
11	NOS	T	1	1–10, G4	Tocilizumab (× 2, D2), siltuximab (× 1, D6)	8–10, G3	Anakinra (D8)	Yes/Yes	CR, MRD-	Alive, relapse (18)
12	NOS	T	90	1–17, G3	Tocilizumab (× 1, D1), DXM (D4), siltuximab (× 1, D5), anakinra (D4)	5–10, G4	DXM (D5), anakinra (D5)	Yes/Yes	CR, MRD-	Alive, CR (17)
13	Common	T	92	1–10, G4	Tocilizumab (× 1, D5), MP (D7), siltuximab (× 1, D8), anakinra (D4)	5–11, G4	MP (D7)	Yes/Yes	PD	DOD (7)
14	Common	T	1.3	1–6, G1	Siltuximab (× 2, D1, D2), DXM (D3), anakinra (D5)	No	No	Yes/-	CR, MRD-	DOD (4)
15	Common	D	87	0–8, G3	Tocilizumab (× 5, D1), MP (D2), siltuximab (× 1, D7), anakinra (D6)	6–9, G3	DXM (D6)	Yes/Yes	CR, MRD-	DOD (21)
16	Common	D	75	5–8, G3	Siltuximab (× 1, D0), MP (D5), anakinra (D4)	8–12, G3	DXM (D8)	Yes/Yes	CR, MRD-	Alive, CR (17)
17	NOS	D	1.2	1–6, G1	Siltuximab (× 1, D1), DXM (D5)	No	No	Yes/-	CR, MRD-	Alive, CR (15)
18	Common	D	80	1–17, G4	Siltuximab (× 2, D1, D7), DXM (D2), anakinra (D1), tocilizumab (× 2, D8)	13–20, G3	DXM (D13)	Yes/Yes	-/Pneumonia	CRM (26 days)
19	Common	D	3.5	1–3, G1	Siltuximab (× 1, D2)	No	No	Ye /Yes	CR, MRD-	Alive, CR (11)

B-ALL, B-cell acute lymphoblastic leukemia; CAR-T, chimeric antigen receptor T cell; NOS, not otherwise specified; F, Female; M, Male; T, tisagenlecleucel; D, tandem CD19/CD22 CAR T-cell therapy; MP, methylprednisolone; MP-HD, methylprednisolone high doses; DXM, dexamethasone; TB, tumor burden; CR, Complete remission; CRi, Complete remission with incomplete hematological recovery; CRS, cytokine release syndrome; ICANS, immune effector cell-associated neurotoxicity syndrome; MRD-, negative measurable residual disease; MOF, multiorgan failure; PD, progression disease; DOD, died of disease; CRM, CAR T-cell related mortality; TRM, transplant-related mortality

Also, 22 patients (34%) of those that received CRS treatment developed ICANS. No statistically significant differences were found in the grade of ICANS between groups (Table 1). Nine of them (40%) received siltuximab, eight of these nine patients (89%) presented a grade ≥ 3 ICANS. The median onset of ICANS occurred at day 10.5 (range 8–13) in patients who received siltuximab as first-line therapy, at day 6 (range 5–8) in patients who received siltuximab for tocilizumab refractory CRS, and at day 8 (range 5–12) in patients who received only tocilizumab. ICANS resolution in the whole siltuximab group was achieved within a median of 4 days (range 2–21) compared with 5 days (range 1–16) in patients that only received tocilizumab. The inflammatory profile of the 5 patients who received siltuximab and the tandem CD19/CD22 CAR T-cell is summarized in Supplemental Table 1.

### Other non-cytokine release syndrome and non-immune effector cell-associated neurotoxicity syndrome toxicities

A total of 18 patients (28%) were diagnosed with an infectious complication. No increased percentage of infections was observed in any of the groups. Among patients that received siltuximab, 2 patients presented bacteremia caused by *Pseudomonas spp.* and *Staphylococcus hominis*; 2 patients experienced viral reactivation (1 cytomegalovirus and 1 BK virus); 1 patient developed *Pneumocystis jirovecii* pneumonia; and 1 patient suffered a reactivation of cytomegalovirus and BK virus along with disseminated tuberculosis. IEC-HS was developed in 11 (17%) patients, half of the those that received siltuximab as first line CRS treatment, not statistical significance. Thirty-seven (57%) patients required admission in pediatric intensive care unit (PICU). Patients that received siltuximab for tocilizumab refractory CRS or as CRS first line treatment presented an 85% and a 67% respectively of PICU admission (p < 0.05). The average length of stay in PICU was 6 days (IQR 5.2), with no statistical significance between groups. The median time to hospital discharge for all patients that received treatment for CRS management was 34 days (IQR 18.75). Delayed ICAHT was observed in 37 (57%) patients that received CRS treatment. Half of those who received tocilizumab and siltuximab as a first-line presented this toxicity in contrast to 11 (85%) of the patients that received siltuximab for tocilizumab refractory CRS (not statistically significance).

### Outcome after chimeric antigen receptor T-cell therapy

In the group of patients who received siltuximab, 3 (16%) died due to CAR T-cell therapy before leukemia evaluation on day 28. The causes of death were 2 multiorgan failures in a refractory CRS, and an IEC-HS with a *Pneumocystis jirovecii* pneumonia (Table 2).

**Fig. 2:**
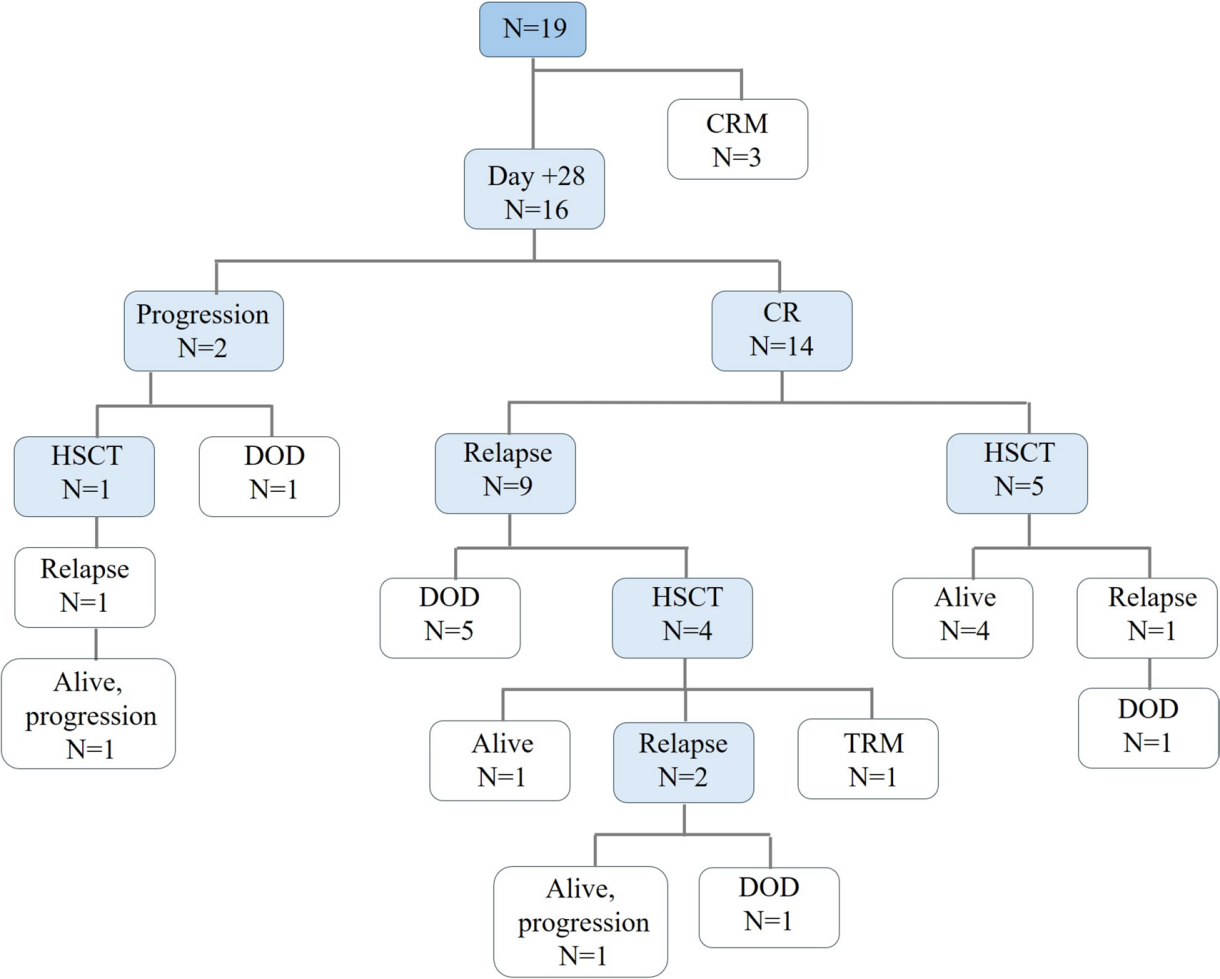
Flow chart of patients who received siltuximab

Fourteen of the 19 (74%) patients that received siltuximab achieved negative minimal residual disease on day 28 (10 of them with incomplete hematological recovery, p < 0.05), and 2 (10%) patients presented disease progression. Five patients (4 who received tandem CD19/CD22 CAR T-cell and 1 who received tisagenlecleucel) of 14 responders (35%), received hematopoietic stem cell transplantation (HSCT) as consolidation treatment after CAR T-cell therapy. Four of these patients (80%), remained alive in complete remission at last follow-up. All 9 responding patients (64%) who did not receive consolidative HSCT relapsed, and only 1 patient (11%) remained alive and free of disease after rescue treatment and HSCT (Fig. 2). With a median follow-up of 10.2 months (IQR 13.2), seven patients (50%) remained alive, two of whom experienced leukemia relapse (28%). Among all patients who received siltuximab after CAR T-cell therapy, the median overall survival (OS) was 10.18 months (95% CI: 4.27–not estimable). For patients who received siltuximab to treat tocilizumab-refractory CRS, the median OS was 7.56 months (95% CI: 3.55–not estimable), while the median OS was not reached for those who received it as first-line therapy.

## Discussion

This study reports the first experience of all Spanish centers accredited for the treatment of pediatric and AYA patients with r/r B-ALL, focusing on the use of siltuximab as part of the treatment for CRS following CAR T-cell therapy.

Prior evidence suggested positive outcomes with the use of siltuximab for the management of CRS or ICANS refractory to tocilizumab and/or steroids in adults with diffuse large B-cell lymphoma (DLBCL) treated with axicabtagene ciloleucel [10]. Additionally, positive outcomes were also reported in adults undergoing first-line therapy targeting CD19 in diffuse large B-cell lymphoma and B-cell maturation antigen in patients with myeloma [11]. Previous data in children are limited to 5 patients after 4-1BB CART19 [8]. Overall, our data reported herein suggest that the use of siltuximab is feasible, even in a high-risk population of patients such as those with a high TB that developed high grade CRS. Additionally, even with the necessary precautions taken due to the heterogeneous and small sample size, we report that the use of siltuximab as a first-line CRS treatment might delay the onset of ICANS. However, larger and prospective studies focused on the onset and duration of this complication in patients treated with siltuximab are needed to demonstrate our first observations.

Limited guidance remains for the optimal management of tocilizumab-refractory CRS; some proposed treatments include drugs such as anakinra (IL-1 receptor antagonist), ruxolitinib (Janus kinase inhibitor), dasatinib (tyrosine kinase inhibitor), emapalumab (anti-IFN-γ), and siltuximab, as well as procedures such as hemofiltration, plasma exchange, and cytokine absorption. It is well known that the most important risk factor for severe CRS in B-ALL patients is the TB prior to lymphodepletion [14]. Therefore, patients with a high risk of severe CRS/ICANS, including those with a TB higher than 5%, might benefit from early interventions [20, 21] or the use of prophylactic drugs [22, 23], as well as from more stable and durable levels of IL-6 antagonist drugs such as siltuximab.

Siltuximab is an approved drug for the treatment of adult patients with multicentric Castleman’s disease who are human immunodeficiency virus and human herpesvirus-8 both negative [6, 7, 24]. Its toxicity profile includes risk of infections due to neutropenia, hypogammaglobulinemia, mild to moderate liver dysfunction and gastrointestinal perforation. In our series, those major toxicities were not observed in patients that received siltuximab, and no significant increase in the incidence of ICAHT, infections or IEC-HS attributable to the use of the drug was reported. Conversely, a higher proportion of severe (ASTCT grade 3–4) CRS/ICANS were noted, which is an expected issue for the group of patients refractory to tocilizumab, requiring a higher percentage of PICU admissions due to the severity of symptoms. For these group of patients, we observed a trend toward a higher frequency of ICAHT, possibly related to the greater inflammatory component, as reflected in the increased severity of CRS/ICANS [25]. Moreover, the weight-adjusted dose of infused cells was higher, which has also been shown to be a risk factor for the development of CRS/ICANS [26]. Interestingly, the apparently increased percentage of patients who received siltuximab and required admission to the PICU could be attributed to the severity of CRS/ICANS rather than the use of siltuximab, which may act as a confounding factor. It is important to note that among the three patients treated with siltuximab who died, two did so in the context of uncontrolled CRS despite the administration of multiple lines of treatment, while the third died due to a respiratory infection secondary to their underlying disease. The use of siltuximab would not inherently justify these fatal events; however, homogeneous studies with a larger number of patients are needed to assess the drug's safety.

Approved dosing for siltuximab is 11 mg/kg single dose every 21 days, and, in theory, this fact could allow for coverage throughout the entire period of risk for CRS and ICANS. In our series, 4 patients received 2 doses of siltuximab at a shorter interval than established because of the severity of toxicities, which suggest that further studies could consider a different posology for CAR T-cell toxicity management.

Like CRS, the pathophysiology of ICANS seems to start with the production of pro-inflammatory cytokines by CAR T-cells and the activation of bystander immune cells such as macrophages in the tumor microenvironment. These inflammatory cytokines such as IL-1β, IL-6, IL-10, the chemokines CXCL8 and CCL2, interferon-γ, GM-CSF r and tumor necrosis factor diffuse into the bloodstream and penetrate the CNS through the blood–brain barrier. ICANS typically occurs after severe CRS, in which the IL-6 cytokine is predominant [27, 28]. Therefore, effective control of CRS might decrease the incidence of ICANS. Our preliminary data suggest a trend to a shorter duration of CRS and a delayed onset of ICANS when siltuximab is used as first line treatment, possibly due to sustained levels of the IL-6 antagonist. Additionally, blocking the IL-6 receptors as tocilizumab does, could paradoxically increase IL-6 levels, potentially triggering and accelerating proinflammatory and endothelial activation, both of which are mechanisms related to the development of ICANS [29]. However, and despite not being statistically significant results, the frequency and severity of ICANS found in the group that received siltuximab, either as treatment for tocilizumab refractory CRS, or as first-line treatment, could be more closely related to patient’s characteristics, TB, CRS grade and CAR T-cell product used (tandem CD19/CD22), rather than to the administration of the drug itself.

Two different types of CAR T-cell constructs for r/r B-ALL treatment have been included in our study. Although they differ in the extracellular recognition domain, they share the same intracellular signaling domains, 4-1BB and CD3ζ, which might not significantly alter signal amplification. However, in other dual approaches, such as bicistronic constructs, coadministration, cotransduction, or sequential infusions, the signaling and amplification could be more potent [32, 33]. Thus, the use of siltuximab could also be considered in patients receiving tandem CD19/CD22 CAR T-cell therapy, in which clinical evidence is showing promise as an alternative to patients with r/r B-ALL who relapse to current CD19 CAR T-cell approaches [34, 35].

The safety and toxicity profile of siltuximab, along with its pharmacokinetics and its direct binding to IL-6 (which prevents the increase in plasma IL-6 levels observed after the administration of other drugs such as tocilizumab) highlight its potential. By inhibiting IL-6’s pro-inflammatory effects, including ICANS, this immunomodulatory drug could be a promising candidate for first-line use in patients at high risk of CRS, such as those with a high leukemia TB.

The present study has some important limitations, including a heterogeneous and small sample size, the use of different CAR T-cell constructs, and its retrospective non-randomized design. Also, patients receiving siltuximab in both, as first-line and rescue treatment for CRS have been included.

In conclusion, refractory CRS remains a significant cause of morbidity and mortality after CAR T-cell therapy. Managing these cases can be particularly challenging and can require aggressive interventions, including additional immunomodulatory therapies. To our knowledge, this study presented the largest cohort reported of pediatric and AYA patients treated with siltuximab for CRS following CAR T-cell therapy for r/r B-ALL. Our findings suggest a tendency toward a safe profile, a shorter duration of CRS, a decreased need for tocilizumab doses, and a later onset of ICANS when siltuximab was used as first-line therapy. Thus, siltuximab might represent an emerging option for the management of CAR T-cell toxicities, and warrants further investigation as a first-line therapy in prospective clinical trials.

## Supplementary Information

Supplementary Material 1.

## Data Availability

No datasets were generated or analysed during the current study.

## Abbreviations

ASTCT: American society for transplantation and cellular therapy
AYAs: Adolescents and young adults
B-ALL: B-cell acute lymphoblastic leukemia
CAR T: Chimeric antigen receptor T cell
CR: Complete remission
CRi: Complete remission with incomplete count recovery
CRM: CAR T-cell related mortality
CRS: Cytokine release syndrome
D: Tandem CD19/CD22 CAR T-cell therapy
DOD: Died of disease
DXM: Dexamethasone
GM-CSF: Granulocyte macrophage colony-stimulating factor
HLH: Hemophagocytic lymphohistiocytosis
HSCT: Hematopoietic stem cell transplantation
ICAHT: Immune effector cell-associated hematotoxicity
ICANS: Immune effector cell-associated neurotoxicity syndrome
IFN: Interferon
IL: Interleukin
IQR: Interquartile range
MOF: Multiorgan failure
MP: Methylprednisolone
MP-HD: Methylprednisolone high dose
MRD-: Negative measurable residual disease
NOS: Not otherwise specified
PD: Progression disease
PICU: Pediatric intensive care unit
r/r: Relapsed/refractory
rCRS: Refractory cytokine release syndrome
rICANS: Refractory immune effector cell-associated neurotoxicity syndrome
SD: Standard deviation
T: Tisagenlecleucel
TB: Tumor Burden
TRM: Transplant-related mortality
