# Supplementary material for: Siltuximab for the treatment of early complications after chimeric antigen receptor T-cell therapy for acute lymphoblastic leukemia in children, adolescents, and young adults
Source: Exp Hematol Oncol. 2025 Apr 2;14:49. doi: 10.1186/s40164-025-00638-3 (PMC11963303; doi:10.1186/s40164-025-00638-3)
Supplement: Supplementary file 1 — Supplementary Material 1. [file 40164_2025_638_MOESM1_ESM.docx]

**Supplemental Table 1.** Summary of the inflammatory profile of the 5 patients who received siltuximab and tandem CD19/CD22 CAR T-cell.

| **TANDEM CD19/CD22**  **PATIENT ID** | **FERRITINE (ng/mL)** | | | | **CRP (mg/L)** | | | | **IL-6 (pg/mL)** | | | |
| --- | --- | --- | --- | --- | --- | --- | --- | --- | --- | --- | --- | --- |
|  | **PRE-SILTUXIMAB**  **INFUSION** | **+24 HOURS** | **+48 HOURS** | **+5**  **DAYS** | **PRE-SILTUXIMAB**  **INFUSION** | **+24 HOURS** | **+48 HOURS** | **+5 DAYS** | **PRE-SILTUXIMAB**  **INFUSION** | **+24 HOURS** | **+48 HOURS** | **+5**  **DAYS** |
| **Patient 15**  (toci-refractory) | 108.635 | 77.373 | 15.493 | 5.706 | 15,3 | 11,7 | 5,5 | 0,5 | 18,1 | 62 | 76,9 | 85,2 |
| **Patient 16**  (first-line) | 1.129 | 1.072 | 1.496 | 3.823 | 2,2 | 10,8 | 12,4 | 7 | 2,9 | 7,4 | 125,8 | 979 |
| **Patient 17**  (first-line) | 1.015 | 905 | 1.229 | 1.990 | 7 | 76,1 | 33,6 | 11,1 | 30,6 | 111,5 | 429,8 | 658 |
| **Patient 18**  (first-line) | 1.132 | 3.411 | 25.401 | 15.154 | 48,8 | 119,7 | 71,1 | 13,3 | 464,3 | 376,5 | 861 | 1.744,7 |
| **Patient 19**  (first-line) | 527 | 419 | 577 | 591 | 89 | 86,5 | 32,9 | 3,4 | 12,9 | 5,9 | 11,3 | 19,6 |
| **MEDIAN** | **1.129** | **1.072** | **1.496** | **3.823** | **15,3** | **76,1** | **32,9** | **7** | **18,1** | **62** | **125,8** | **658** |
| **IQR** | **117** | **2506** | **14.264** | **3.716** | **41,8** | **74,8** | **21,2** | **7,7** | **17,7** | **104,1** | **352,9** | **893,8** |

IQR: interquantile range
